# The Clinical Characters Associated with Certain Types of Degeneracy, with Remarks on Treatment

**Published:** 1900-10-20

**Authors:** W. Lloyd Andriezen

**Affiliations:** late Deputy-Superintendent of the Metropolitan Asylum at Darenth, and formerly Assistant Medical Officer at the West Riding Asylum, Yorkshire


### Hospital Clinics and Medical Progress.

### THE CLINICAL CHARACTERS ASSOCIATED WITH CERTAIN TYPES OF DEGENERACY, WITH REMARKS ON TREATMENT.

By W. Lloyd Andriezen, M.D. (Lond.), late Deputy-Superintendent of the Metropolitan Asylum at Darenth, and formerly Assistant Medical Officer at the West Riding Asylum, Yorkshire.

The medical practitioner is not unfrequently consulted regarding a certain type of cases in which the patient does not exhibit an amount of mental defect amounting to idiocy or low-grade imbecility, and yet is not the subject of mental and moral alienation sufficient to render his certification and sequestration in a lunatic asylum necessary. Some of these cases are complicated with epilepsy or hysteria or other grave neurosis, and others show a more distinct tendency to dangerous and criminal impulses, yet they all have certain "degenerative" characteristics in common, which mark them out as members of one family—the group of degenerates in the sense in which that epithet is used in the classical works of Morel and Magnan. The clinical signs and symptoms which constitute the marks or "stigmata" of degeneracy are numerous and varied. They are external indications of the taint within, and may assume various forms; but they should always be distinguished from accidentical deformities, such as intra-uterine accidents to the limbs, e.g. amputation, or the traumatisms sustained in parturition and infancy.

The following cases, which have come under observation both in asylum experience and in private practice, will serve to illustrate some of the facts more clearly, and will serve to show what methods should be pursued in regard to treatment.

*Case* 1.—M. H. H., male adolescent. Is stunted in growth, slight in physique, and has large, flappy, outstanding ears (a stigma of degeneracy). Is somewhat weak-minded, and obstinately refuses to have his hair cut or his beard (of which there is a moderate growth) cropped. His parents and sister, whom I have seen, are themselves dull in mind, stunted in body, ignorant, and bigoted. The patient readily develops delusions that he is being persecuted by others (which is not true), and these delusions are sympathetically shared in by his relatives.

The patient is best suited for detention in an industrial colony where he could receive suitable education and training so as to make him relatively useful and industrially productive. If delusions become dangerous, he would require asylum treatment.

*Case* 2.—T. C., male adult. Has a small head (submicrocephalic) with narrow elongated foxy face and small, obliquely set, reddish, ferrety-looking eyes. Is weak-minded, but not imbecile, and is the subject of homosexual perversion. Under supervision he is industrious and useful.

A case like the above would probably benefit best in a "Special Institution for Degenerates," such as has been recommended by Professor Morel of Belgium (*Journal of Mental Science*, October, 1899).

*Case* 3.—J. C., male adult. Belongs to the class described by Lombroso and others as "criminal by organisation" and allied to the epileptic and the alcoholic. He is subject at times to violent maniacal excitement and impulses, and is then very destructive to clothing and property. He had been in prison (seven days) for having been "drunk and disorderly," and is said to have, during one of his fits of excitement, attacked his warders. He admits that he has no self-control when he takes a little beer. He has been in an asylum repeatedly owing to his maniacal outbreaks. He has a shifty, furtive, hang-dog look and exhibits facial muscular twitchings like those of chronic alcoholism. He is full of plausibility and deception, and promises to exercise self-control in the future, which his past and present records completely negative. Alcoholic habits have made him worse of late, and his promises of reform are vain and worthless. He suffers occasionally from furor, like that of epilepsy. The proper institution for such a case as the above is the asylum, as he would be too troublesome and unmanageable in a farm or industrial colony.

*Case* 4.—T. C., young male adult. Is of the criminal and degenerate class, prone to develop delusions and requiring asylum care and sequestration. He is not epileptic. He had been a soldier in India, where he had committed various delinquencies, for which he suffered imprisonment; he also had had the cat-o'-nine-tails. He used to drink carelessly. Since his arrival home he has been low-spirited; he fancied that dogs and cats came to the door of his room (a delusion), throttled his sister, and kicked his mother on the stomach for remonstrating. The patient is tall, thin, and lanky, able-bodied but inclined to idleness specious and prolix in talk, hiding his faults with much verbiage; has a low, contracted forehead scored with wrinkles (a mark of criminal degeneracy—*vide* Havelock Ellis, *The Criminal*, p. 71–2), high cheek bones, a thick fleshy nose, and a prominent lower jaw—a physiognomy showing on the whole many stigmata of degeneracy. Is of a skulking and sinister aspect, with shifty countenance and is emotional (easily excited to anger and hysterical tears), with little self-control. Disposition morose and suspicious. There are evidences of old gonorrhœa and of bubo in the groins.

The above case, owing to the marked mental alienation and the tendency to violent and dangerous impulses, finds suitable treatment only as an asylum patient.

*Case* 5.—H. W., male youth, pubescent. Features pleasing, apart from a moderate degree of facial asymmetry. At rare intervals has epileptic fits. Is frequently impulsive and passionate, destructive and given to throwing things through the windows in a passion. Sexually precocious and perverted in instinct, and given to masturbation as a child. Well-formed, smooth and supple in body and limbs. Untruthful, plausible, full of low cunning.

The above case can scarcely be suitable for an epileptic farm colony owing to the occurrence of destructive and violent outbreaks and the marked sexual depravity present. He would be more suitable for an institution like that described by Professor Morel for degenerates.

*Case* 6.—E. C., young female, pubescent. Has *petit mal* at rare intervals. Weak-minded as regards *viva voce*, instruction, but reads greedily almost everything. Is excessively ugly in features. Has attacks of bad temper very often and is then refractory, sullen, and insolent towards her mistress. Has marked amnesia at such times. Is considered likely to develop epileptic fits more frequently when she becomes a little older.

A case such as the above would be suitable for private care if means permit, or as an inmate of an industrial colony for the weak-minded. Such a girl without special supervision might easily go wrong and become the mother of degenerate and illegitimate children.

*Case* 7.—A. S., female adolescent. The illegitimate daughter of a weak-minded woman, who has had two other illegitimate (female) children. Is weak-minded but possessed of considerable low cunning, and has incorrigible thievish and lying habits. Five years ago, at the age of fourteen, she suffered the embraces of a boy of sixteen and contracted gonorrhœa.

Has little or no idea of morality, and is likely to become the mother of degenerate children. Is at large in domestic service, though she would be more suitable in a farm colony for the weak-minded. Is not epileptic.

*Case* 8.—G. W., male pubescent youth. His habits from childhood have been those of a street arab, petty pilferer, vagrant, and he was convicted of vagrancy and imprisoned for a month. He was then employed in a coffee-house, which he left under the delusion that enemies were following him. He tramped a considerable distance, and when in prison he saw his imaginary persecutors getting in dressed as prisoners and having fits to frighten him. There is a marked taint of delusional insanity. Under the influence of delusions that his persecutors were going to kill him he became violent and broke windows. He has marked facial asymmetry, and the nose is considerably inclined to one side, while the lips and angle of the mouth also show asymmetry and are drawn up to the left in smiling. The type of physiognomy is sharp and cunning, but of the imbecile character. The left orbital fossa is narrower than the right. He has had right otorrhœa since childhood. There is a congenital defect of will (aboulia), he is puerile, maniacal at times, and again weeps and whines childishly. Under improved conditions and proper treatment he has progressed satisfactorily, having grown more cheerful, willing and industrious and less given to impulse. The delusions have disappeared, though liable to recurrence.

The above case requires asylum treatment, owing to the nature of the delusions and their tendency to produce maniacal attacks. Recovery in such cases is only partial, and the ultimate prognosis is bad, owing to the presence of grave mental and bodily stigmata of degeneracy, and the liability to recurrent exacerbations, as was pointed out especially by Magnan to be a characteristic of such cases.

